# Wound inflammation post-orchiectomy affects the social dynamic of Nelore bulls

**DOI:** 10.1186/s12917-023-03638-9

**Published:** 2023-07-15

**Authors:** Caique Marques Marcelino, Pedro Henrique Esteves Trindade, Henry David Mogollón García, Antonio Guilherme Roncada Pupulim, Cyntia Ludovico Martins, Guilherme Rizzoto, Francisco Teixeira-Neto, Fernanda Macitelli, John Patrick Kastelic, João Carlos Pinheiro Ferreira

**Affiliations:** 1 Department of Veterinary Surgery and Animal Reproduction, School of Veterinary Medicine and Animal Science, São Paulo State University, Street Prof. Dr. Walter Maurício Correa, Botucatu, 18618-681 Brazil; 2 Department of Animal Production and Preventive Veterinary Medine, School of Veterinary Medicine and Animal Science, São Paulo State University, Street Dr. José Barbosa de Barros, Botucatu, 18610-307 Brazil; 3 Institute of Health Sciences, Mato Grosso Federal University, Av. Alezandre Ferronato, Sinop, 78550-728 Brazil; 4 Department of Production Animal Health, Faculty of Veterinary Medicine, University of Calgary, 3330 Hospital Dr NW, Calgary, Canada

**Keywords:** Animal welfare, Cattle, Dominance, Hierarchy, Pain

## Abstract

**Background:**

Confinement of cattle imposes spatial restrictions and predisposes to aversive social encounters that can lead to contusions, wounds, pain, stress, fright, and reduced productivity. Although endogenous testosterone concentrations are linked to agonistic dominance behaviors in males, it is unknown whether decreased blood testosterone concentrations after castration alter social hierarchy rank in Nelore bulls. Therefore, in this study, we investigated the impact of the surgical would inflammation post-orchiectomy on social dynamics in a group of Nelore bulls (*Bos indicus*). Fourteen Nelore (Bos indicus) bulls were castrated and assessed pre- and post-surgically. Parameters evaluated were agonistic (mounting, headbutting, and fighting) and affiliative (head-play) behavior, plasma testosterone concentrations, average daily weight gain (ADG), and a score for severity of post-surgical infection. Exploratory statistics included social network analysis (SNA), hierarchy rank delta (Δ), and principal component analysis (PCA). Furthermore, statistical inferences included the Wilcoxon test, multiple logistic regression models, and Spearman's correlation.

**Results:**

The social dynamic of Nelore bulls was modified after castration based on the findings of the SNA and the PCA. The moderate correlation between the postoperative inflammation level with the Δ, and the significant effect of this level in the logistic model post-castration were partially attributed to effects of pain on social relations.

**Conclusions:**

Our findings suggest the severity of post-surgical inflammation, which has an association with pain intensity, was closely associated with changes in the social hierarchy.

**Supplementary Information:**

The online version contains supplementary material available at 10.1186/s12917-023-03638-9.

## Background

In the past few decades, there is increasing worldwide concern regarding production animal welfare [1]. Welfare status can be impacted by various stimuli that modulate the homeostasis of biological functions affecting physiological and mental states in animals [2–4]. In that regard, several factors involved in beef cattle production may reduce bovine welfare, including intensification of animal production in a confinement system [5].

Confinement imposes spatial restrictions, resulting in competitive encounters and violation of individual spaces; consequently, there is intensification of agonistic social behaviors (e.g., pushing, mounting, fighting, and headbutting) [6–10], as well as a reduction in affiliative behaviors (e.g., head-play and grooming) [11]. Furthermore, prior to entering confinement, cattle are usually sorted by weight, age, and sex and placed in a pen with cattle from various origins [12]. Mixing of animal groups can also occur at the end of the confinement period to regroup cattle from other pens that were not sent to slaughter. In both cases, regrouping modifies the primary social order [13, 14], leading to instability in the social ranking of the herd and increasing agonistic interactions to establish a new hierarchy [6, 7, 9, 10]. These agonistic social interactions can cause contusions, physical lesions, and mental states of pain, stress, and fear, reducing welfare status [15] and cattle productivity [7, 11].

Exacerbated displays of agonistic behavior in males are apparently modulated by endogenous testosterone [16]. In non-human primates [17–20], rats [21] and guinea pigs [22] alpha males (non-castrated) had higher testosterone concentrations than subordinate males. In cattle, testosterone propionate can modulate some behavioral patterns, e.g., thyroidectomized *Bos taurus* males recovered physical activity and sexual behavior [23]. Similarly, in heifers, testosterone propionate leads to dominance in the social hierarchy rank compared to untreated animals, with no increase in agonistic interactions, with the previous dominance restored after treatment ended [10].

Castration of bulls has been associated with easier and safer animal handling and reductions in lesions and aggressive behavior [24–29]. However, whether post-castration reductions in testosterone concentrations and postoperative consequences affect social interactions in cattle is unknown. Our objective was to investigate the impact of surgical castration on social dynamics in a group of Nelore bulls.

## Results

**Fig. 1:**
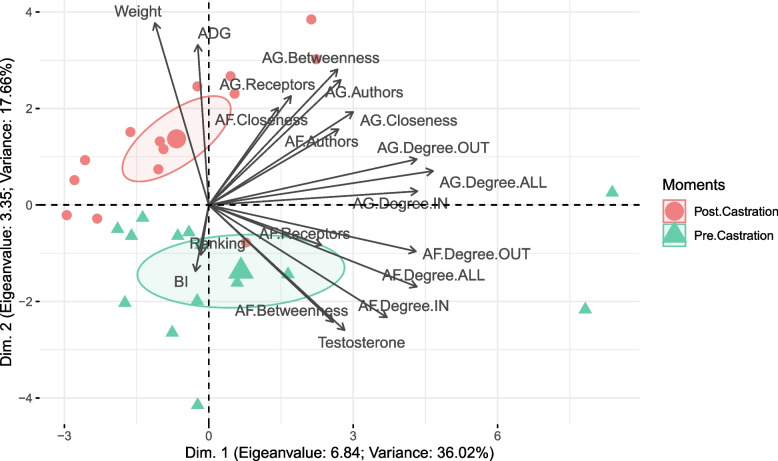
Principal component analysis of Nelore bulls before and after castration. (AG = agonistic behavior; AF = affiliative behavior; Betweenness = parameter that quantifies the number of times that a bull acts as a bridge throughout the shortest path between two other bulls; Closeness = parameter that quantifies the proximity that a bull has with every bull in the social network; Degree ALL = total number of social interactions (authoring and receiving); Degree IN = number of receivals in the social interaction; Degree OUT = number of receivals in the social interaction; Authors = score of bulls with higher authoring and smaller receiving index of social interactions; Receptors = score of bulls with smaller authoring and higher receiving index in the social interactions; ADG = average daily weight gain; and BI = balance index)

Regarding social network analysis (SNA), numbers of connections and reciprocity of the agonistic and affiliative interactions in the social networks (Table 1), as well as separation of the ellipses corresponding to the pre-castration period (majorly positioned in the bottom-right quadrant) and post-castration (majorly positioned in the top-left quadrant) of the principal component analysis (PCA) (Fig. 1). These exploratory results suggest a modification in the hierarchical structure when comparing pre- *vs* post-castration moments.

**Table 1:** Descriptive parameters for analysis of the social network built for agonistic and affiliative behaviors before and after castration of Nelore bulls

Parameters	Agonistic		Affiliative	
	Pre-castration	Post-castration	Pre-castration	Post-castration
Density	0.54	0.34	0.39	0.19
Asym	64	53	15	17
NULL	10	34	33	28
Diameter	4	9	4	3
Connections	98	61	51	17
Reciprocity	0.35	0.13	0.71	0.00

Density = percentage of existent social interaction in the social network; Asym = parameter that estimates the asymmetry degree considering the direction of the paths (interactions); NULL = number of possible interactions between bull that didn't take place in the social network; Diameter = diameter of the social network; Connections = number of existent interactions in the social network; and Reciprocity = probability of the bulls to display mutual social interactions

**Fig. 2:**
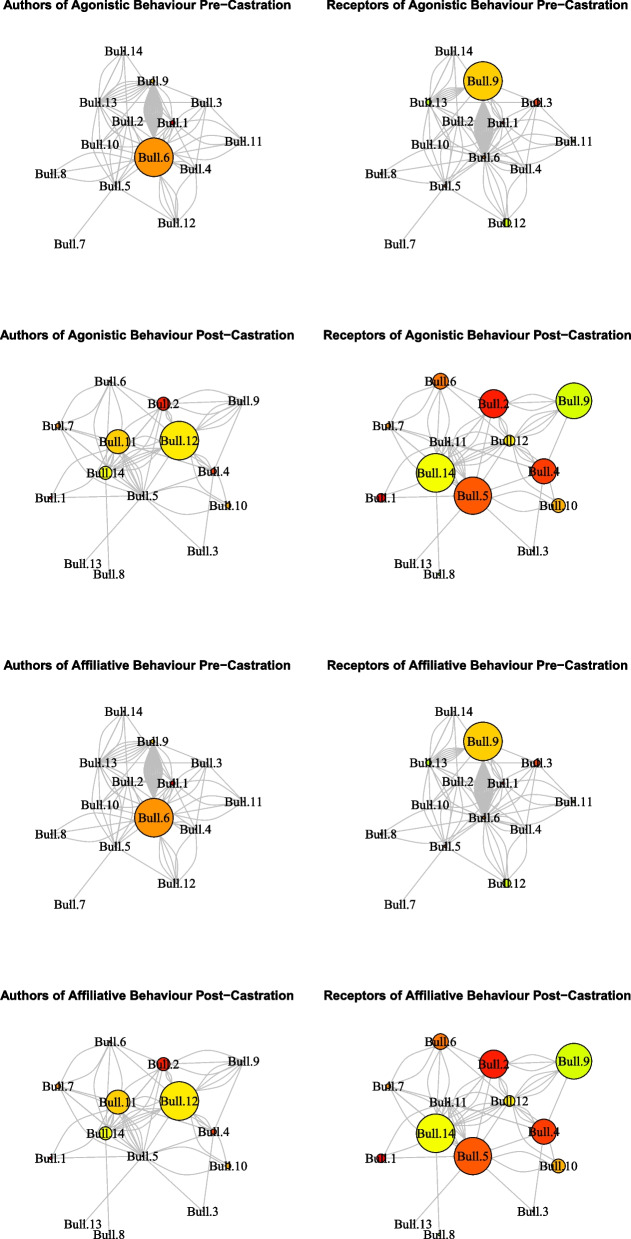
Representation of the net analysis built with agonistic and affiliative behaviors in Nelore bulls before and after castration. (Bulls are represented by nodes (circles). The diameter of the nodes is proportional to the score of the author or the receptor. Lines indicate the interaction between each bull. Author score = score of bulls with higher authoring and smaller receiving index of social interactions; Receptor score = score of bulls with smaller authoring and higher receiving index in social interactions)

On the first dimension of the PCA, the loading value of testosterone concentrations (0.57) indicated it had limited association with other variables and only limited contributions in the dimension (Table S2). The SNA indicated an inversion of the individual social conduct for bulls that downgraded the scores of the authorship of agonistic (Bulls 1, 6, and 9) and/or affiliative behaviors (bulls 5, 7, 11, and 12) (Fig. 2).

Comparisons between moments indicated that the group of cattle had increased tolerance in receiving agonistic behaviors (ReceptorsAG). Furthermore, the interactive capacity between the whole group for affiliative behaviors (ClosenessAF), body weight and ADG, also a decreased total number of affiliative interactions (Degree ALLAF), tolerance to receive (Degree INAF) affiliative behaviors, testosterone, and BI post castration (Table 2).

**Table 2:** Median (minimum; maximum) of social interaction parameters of the social network analysis for agonistic (AG) and affiliative (AF) behaviors, balance index (BI), testosterone concentration, weight, and average daily weight gain (ADG) before and after castration of Nelore bulls

Parameters	Pre-castration	Post-castration
	Median (min; max)	Median (min; max)
Betweenness_AG_	7.62 (0.00; 66.21)	14.96 (0.00; 42.70)
Closeness_AG_	0.04 (0.03; 0.06)	0.04 (0.031; 0.06)
Degree ALL_AG_	8.00 (1.00; 55.00)	7.50 (1.00; 21.00)
Degree IN_AG_	5.00 (0.00; 24.00)	3.50 (1.00; 13.00)
Degree OUT_AG_	4.00 (0.00; 31.00)	2.5 (0.00; 18.00)
Authors_AG_	0.01 (2.15^–18^; 1.00)	0.10 (6.61^–18^; 1.00)
Receptors_AG_	0.05 (1.00^–26^; 1.00)^b^	0.32 (5.36^–3^; 1.00)^a^
Betweenness_AF_	0.66 (0.00; 13.74)	0.00 (0.00; 8.00)
Closeness_AF_	0.02 (0.00; 0.03)^b^	0.04 (0.00; 0.08)^a^
Degree ALL_AF_	5.00 (0.00; 31.00)^a^	1.50 (0.00; 10.00)^b^
Degree IN_AF_	3.50 (0.00; 12.00)^a^	0.00 (0.00; 9.00)^b^
Degree OUT_AF_	2.00 (0.00; 19.00)	1.00 (0.00; 5.00)
Authors_AF_	0.07 (4.83^–18^; 1.00)	0.16 (1.83^–17^; 1.00)
Receptors_AF_	0.16 (1.96^–3^; 1.00)	0.09 (8.39^–18^; 1.00)
Testosterone	3,529.80 (643.90; 15,600.60)^a^	3.90 (3.90; 3.90)^b^
Weight	432.00 (386.00; 483.00)^b^	521.00 (464.00; 592.00)^a^
ADG	-1.76 (-6.56; -0.92)^b^	0.47 (-0.53; 1.39)^a^
BI	1.24 (2.50^–10^; 33.50)^a^	0.03 (5.00^–11^; 8.96)^b^

Betweenness = parameter that quantifies the number of times that a bull acts as a bridge throughout the shortest path between two other bulls; Closeness = parameter that quantifies the proximity that a bull has with every bull in the social network; Degree ALL = total number of social interactions (authoring and receiving); Degree IN = number of receivals in the social interaction; Degree OUT = number of receivals in the social interaction; Authors = score of bulls with higher authoring and smaller receiving index of social interactions; Receptors = score of bulls with smaller authoring and higher receiving index in the social interactions; ADG = average daily weight gain; and BI = balance index. Different letters between the columns indicate a difference (*P* < 0.05), with a > b

**Fig. 3:**
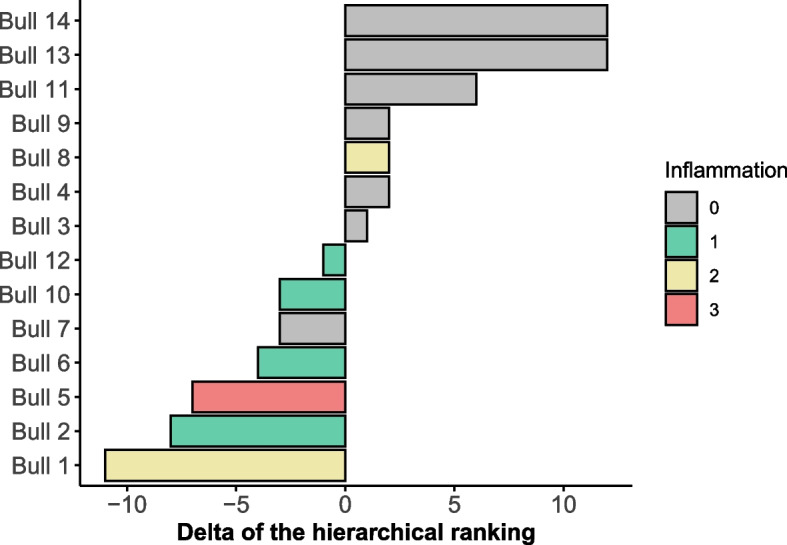
Delta (Δ) of the ranking hierarchy and post-castration inflammation levels in Nelore bulls. (Delta [Δ] is the subtraction of post-castration hierarchy rank of one animal from the pre-castration rank. Inflammation level score was defined as 0, 1, 2 and 3 [corresponding to absence, mild, moderate and severe inflammation, respectively])

In the post-castration evaluation, four bulls had a mild inflammation of their surgical wound (Bulls 2, 6, 7 and 12), two had a moderate inflammation (Bulls 10 and 13), and one had severe inflammation (Bull 5) (Table S1). Only the inflammation level had significance in the experimental design to explain upgrading or downgrading in the post-castration ranking (Table 3); consequently, all animals with negative delta (Δ) in the ranking had some degree of post-surgical inflammation, with Bull 7 being the only exception (Fig. 3). The Δ of the ranking had a moderate negative correlation (rho =—0.68; *P* = 0.0073) with level of inflammation. However, there was no difference for post-castration average daily weight gain (ADG) between bulls with Δ negative or Δ positive ranking (respectively, mean and range 0.46: 0.00 – 0.99 and 0.53: -0.53 – 1.39; *P* = 0.4036).

**Table 3:** Estimated value, standard deviation, z-value, and *p*-value of the logistic regression before and after castration of Nelore bulls

Model		Estimate	Standard Error	Z-value	*P*-value
Pre-castration	(Intercept)	-0.06544	10.42763	-0.006	0.995
	Weight	-0.00152	0.023175	-0.066	0.948
	ADG	0.002976	0.420582	0.007	0.994
	Testosterone	0.000146	0.000129	1.133	0.257
Post-castration	(Intercept)	-20.5786	13.834	-1.488	0.1369
	Weight	0.03827	0.02492	1.536	0.1246
	ADG	-1.02773	2.12802	-0.483	0.6291
	Inflammation score	3.21203	1.57587	2.038	0.0415
	Surgery order	-0.18789	0.2756	-0.682	0.4954

## Discussion

The novelty of this study was the investigation of effects of surgical castration on social dynamics of bulls. In this study, the structure of the social hierarchy ranking of the Nelore bulls changed after the castration. There was no clear influence of testosterone in this phenomenon. However, there were strong indications connecting the level of post-castration inflammation with changes in the social hierarchy ranking, perhaps related to pain level. Therefore, our findings indicated the need for further studies investigating the relationship between pain relief and maintenance of the social hierarchy rank to promote animal welfare and productivity.

In our study, the increase in the tolerance to receive agonistic behaviors, the reduction of reciprocity of social interactions, and lower density in the social network after castration were interpreted as responses to reduced aggressivity. This was expected, as during the last four decades, studies with *B. taurus* and *B. indicus* bulls have demonstrated that castration reduces the display and repertoire of social behaviors related to aggressivity, thereby improving cattle welfare [24–29].

Another novelty of this study was that in addition to the reduction in the post-castration agonistic interactions being a clear improvement in welfare [24], organization of the social hierarchy, as demonstrated by the SNA, was strongly modified. Social organization is based on dominant-subordinate relationships, established through fighting, pursuing, pushing, and headbutting [6, 7, 9]. Therefore, it was plausible that these animals intensified their agonistic interactions until a new hierarchy was established, determined by reductions in aggressive social encounters [30], as observed after castration in this study.

Interestingly, the process of establishing a social hierarchy can reduce cattle welfare, including potential lesions, pain, stress, and fear [15], in addition to reductions in productivity [7, 11]. However, in this study, there was no statistical difference in the ADG of bulls that upgraded or downgraded in the hierarchy ranking, nor any significant effect of ADG or weight in the model of the Δ of the ranking, contrary to previous reports. This can be partially explained by the limited number of cattle used in the study (*n* = 14) and feeding a maintenance diet. In 37 Friesian bulls, there was an effect of social ranking on ADG [11], whereas Jersey/Holstein heifers with a lower classification in the social hierarchy had less access to feed [31].

We hypothesized that after surgical castration testosterone reductions affect social hierarchy rank in Nelore bulls. For non-human primates [17–20], rats [21], guinea pigs [22], and bulls [23], dominant males have higher testosterone concentrations than their subordinates. Conversely, the limited association between testosterone concentrations and other variables (loading value in the first dimension of the PCA) and the lack of significance (*p* > 0.05) in the modeling of the Δ of the pre-castration ranking indicated the absence of an evident influence of testosterone in modulation of social hierarchy, as observed in rats [32–34]. Furthermore, it was recently reported that the dominance hierarchy can be very sensitive to stress factors in the social context. In that regard, previous stress experiences, including during neuroendocrine development, can determine future social dynamics [35–38]. The controversy of the previous studies highlighted the complexity of the hierarchical structure and challenged the paradigm that endogenous testosterone is the singular key to understanding the social hierarchy in a gregarious species.

Pain can be a stress-inducing factor capable of modulating social interactions in laboratory animals [39]. For example, in alpha male rabbits, acute pain induced by intra-plantar application of formalin reduced displays of aggressive behavior [40]. Conversely, mice treated with either formalin (intra-plantar) or acetic acid (intra-peritoneal) had increased aggressive behavior after 7 d, indicating that long-lasting pain can impact 'social memory' (the individual perception of hierarchy position of each animal in the group), causing instability in the group's social organization [41]. Additionally, alpha male mice with induced neuropathic pain downgraded their social position compared to males not exposed to pain [42].

In the present study, modifications in the social ranking of bulls post-castration, the moderate correlation between the surgical wound inflammation post-orchiectomy level with the Δ of the ranking, and the significant effect of this level in the logistic model post-castration were partially attributed to effects of pain on social relations. We inferred that pain originating from surgical wound inflammation post-orchiectomy modified hierarchy. Furthermore, another perspective is that the treatment of such surgical wounds post castration could have increased stress, potentializing the indisposition towards socialization and impairing its social position, since most bulls in this study with any degree of inflammation downgraded their position in social rank. Perhaps maintenance of the social hierarchy is dynamic and sensitive to the perceptible opportunities for each bull, allowing upgrading in social ranking in the face of stressful factors impacting the group. This can be understood as an adaptive value for species' survival, based on the Darwinian theory. In summary, there was a modification in social hierarchy in a medium-term after the would inflammatory episode; however, the short-term response needs to be evaluated to improve understanding of the influence of pain on social hierarchy of the bulls. This is important, since several events may have occurred between the pre- and post-castration behavioral evaluations, which were not documented in our study, and which should be investigated in the future. Our results represent the first step in the investigation about surgical castration and social dynamics of bulls.

The present study was not without limitations. Despite only a limited number of animals used (*n* = 14), they displayed social interactions sufficiently capable of differentiating pre- and post-castration social hierarchy. Plausibly, a survey with more bulls, such as in commercial feedlots, would result in an even further increase in social interactions. Lastly, to castrate only three bulls every 15 d could have impacted our study's findings; however, the logistic model of the Δ of the ranking post-castration did not indicate any significant effects related to the sequence in which the bulls were castrated. We emphasize that this was an opportunistic study, and the experimental design employed here was limited by the main experiment with the same animals [43]. In addition, the choice of design also supported the 4 R's concept [44, 45] to reduce the number of animals used in research. Furthermore, bulls were not castrated on the same day; therefore, the study's experimental design emulated, to an extent, a real field scenario.

In a practical way, our results suggested that intensification of agonistic social encounters was affected by surgical wound inflammation post-orchiectomy. This finding strengthens the importance of diagnosis and treatment of pain to minimize social instability of the group; regardless, pain relief is essential to reduce suffering [46]. Therefore, adequate analgesia after any painful procedure during confinement is of fundamental importance. Furthermore, such actions are relevant in reducing the intensification of agonistic behaviors to mitigate economic losses and reductions in welfare status.

## Conclusions

In this study, a change in the social hierarchy order of Nelore bulls after surgical castration was apparently associated with surgical wound inflammation post-orchiectomy.

## Methods

The Animal Care Committee of the host institution approved this study (ID 029/2018). A fortunate opportunity of another study from our team [43] taking place at our institution facilitated analysis of the behavioral data of the same group of bulls; furthermore, this approach supported the 4R's (reduce, replace, refine and, respect; [44, 45]).

### Animals, handling, and procedures

Fourteen Nelore bulls (B. indicus; ~ 2 y; ~ 400 kg) were used in the study, all derived from the same commercial herd. After arrival at our institution, the bulls were individually restrained in a cattle handling chute, body weight recorded and a 10-mL blood sample collected from coccygeal vessels into heparinized tubes (DsysLAB®, Curitiba, PR, Brasil) that were centrifuged (2,000 × g for 5 min) and plasma removed and frozen (-20 ºC) pending a testosterone assay.

The bulls were housed together in one outdoor pen (10 × 15 m; 150 m2) with 1.25 m of feeder area and ad libitum water provided through an automatic auto-fill water tank (1,500 L; diameter, 1.5 m). For acclimatization, for 15 d after arrival, there was minimal human contact, only providing feed and cleaning the feeder and water tank. Twice daily, the bulls were fed a maintenance diet comprised of corn silage (63.18%), cornmeal (20.58%), cottonseed meal (10.77%), sugarcane bagasse (4.11%), urea (0.69%), limestone (0.5%) and mineral salt (0.17%).

After 15-d acclimatization, bulls were weighed a second time, average daily weight gain (ADG) calculated, and the data related to the pre-castration period. Throughout the subsequent 5-d interval, behavioral assessments were performed (described below); on the first day, the animals underwent general anesthesia, were exposed to testicular heat stress (3 h at ≤ 40 ºC) and castrated immediately thereafter, as described [43]. Three bulls were submitted to these procedures every two weeks until all were castrated. The order of operation was based on the scrotal circumference (descend order), as required by the principal experiment that bulls were used [43].

Prophylactic antimicrobial therapy was administered before castration (1.1 mg/kg of ceftiofur IM, Cef50®, União Química Farmacêutica Nacional S/A, Embu-Guaçu, SP, Brazil). Furthermore, a nonsteroidal anti-inflammatory and analgesic treatment was given (1.1 mg/kg of flunixin meglumine IV, Banamine®, MSD Saúde Animal, Cruzeiro, SP, Brazil). Post-surgical inflammations were treated symptomatically. The wound was cleaned with water and antiseptic solution (Riodeine Dermatologico Suave Tópico®, Rioquímica, São José do Rio Preto, SP, Brazil) followed by application of insect repellent (Tanidil, Bayer®, São Paulo, SP, Brazil) and an agent to promote cicatrisation (Unguento Chemitec®, Chemitec Agro-Veterinária, São Paulo, SP, Brazil). Furthermore, the animals were treated with 12,000 IU/kg of benzylpenicillin benzathine, benzylpenicillin procaine, benzylpenicillin potassium IM, and 5 mg/kg disulfate dihydrostreptomycin disulfate base and streptomycin base IM (Penta-biotic 6,000,000 UI®, Zoetis, São Paulo, SP, Brazil). Furthermore, they were weighed twice (3 and 20 d) after the last castration, and post-surgical ADG was calculated. Lastly, at 21 d post castration, a blood sample was collected (as described above) and used to determine post-castration testosterone concentration.

After the end of the experimental period, all animals were incorporated into the faculty herd.

### Behavioral registers

After the 15-d acclimatization period, the social hierarchy pre-castration was determined at ~ 07:30 am, based on the record of the frequencies of affiliative and agonistic interactions through the application of the ethogram described in Table 4, identifying animals as authors and receptors. This behavioral observation was performed in loco continuously by the focal animal sampling method [47] for 2 h after the first feeding of concentrate of the day, for five consecutive days by the same evaluator (CMM). This evaluator was a member of the team management of the bulls, taking care their since arrival, then the bulls were adapted to the presence of this human. The choice of the behavioral observation period was based on a previous study, in which 88% of the agonistic behaviors occurred during feeding due to competition for resources [48]. To characterize post-castration social hierarchy, 43 d after the castration of the last bull, behavioral observations were repeated, as described above.

**Table 4:** Ethogram of affiliative and agonistic social interaction frequency used to characterize social hierarchy of Nelore bulls

Category	Behaviour	Description
Agonistic	Mount	A male (author) raises the thoracic region of its body close to another male, flexes the thoracic members, and can stand on the rump of the other male (receptor) to perform the mount; with possibility of penetration or attempt when the receptor moves to any direction to avoid being mounted
	Fight	An animal (author) presses the forehead of another animal (receptor) with its own; both animals apply forward pressure in a vigorous, aggressive manner for an interval, with possible bumps and kicks
	Headbutt	An animal (author) uses its head to hit the other bull (receptor), forcing the receptor to move at least one step in any direction
Affiliative	Head-play	An animal (author) touches its head to the forehead of another animal (receptor), both animals apply forward non-vigorous pressure to the contact point

### Postoperative inflammation

The degree of surgical wound inflammation post-orchiectomy was evaluated by the same evaluator (HDGM) using a four-level score. Scoring was based on clinical evaluation of regional edema and swelling and the presence of haemorrhage or myiasis, and graded as follows: 0, 1, 2 and 3, representing no, mild, moderate, and severe inflammation, respectively.

### Plasma testosterone concentrations

Plasma testosterone concentrations were measured with a commercial ELISA kit (Testosterone ELISA Kit®; #582701 – Cayman Chemical, Ann Arbor, MI, USA), with a 3.9 pg/mL sensitivity. Pre-castration samples were diluted 1:24 with the ELISA Buffer included in the kit. Duplicates were performed for the first and last samples measured; the intra-assay CV was 7.4%. Although no dilution was performed, the samples did not reach the minimum limit of identification. Therefore, the minimum detectable level indicated by the company (3.9 pg/mL) was used.

### Statistical analyses

All statistical analyses were performed by the same data scientist (PHET), using the software R with integrated developing system RStudio (Version 4.0.2 (2020–06-22), RStudio, Inc.). Various packages and functions were used in the format 'function{package}' according to the programming language in R. An α level of 5% was used for all tests. For the analyses below, bodyweight considered was the last bodyweight of either the pre- or post-castration intervals.

The ADG was calculated using the following equation:

$$ADG=\frac{\left(Wf-Wi\right)}{d}$$

ADG = average daily gain; Wf = body weight at the end of the experiment; Wi = body weight at the beginning of the experiment; d = average interval (d) between the two weights.

A priori, the inter-relation between bulls was analyzed with exploratory statistical techniques. The frequency of agonistic behaviors was the sum of the frequency of mounts, fights, and headbutts. Four social network analyses (SNA) were performed ('as_data_frame{igraph}'), one for each behavioral category (agonistic and affiliative) in pre- and post-castration moments. The following parameters were extracted: asymmetry; diameter; connections; reciprocity; betweenness, closeness, degree (all, in, out), hub score (authors), and authorities score (receptors).

Furthermore, based on data extracted from the SNA, the balance index (BI) was calculated based on a previous report in dairy bulls (Foris et al., 2019), using the following equation:

$$BI=\frac{\frac{\mathrm{IN}affiliative +F1}{\mathrm{IN}agonistic +F2}}{\frac{\mathrm{OUT}affiliative +F1}{\mathrm{OUT}agonistic +F2}}$$

INaffiliative: frequency that the animal was the receptor of affiliative behavior; INagonistic: frequency that the animal was the receptor of agonistic behavior OUTaffiliative: frequency that the animal was the author of affiliative behavior; OUTagonistic: frequency that the animal was the author of agonistic behavior; F1: correction factor of 0,005; F2: correction factor of 0,001. A small value was used as a correction factor to avoid a zero quotient in the absence of behavior display (adapted from [49]).

As indicated in previous studies that demonstrated the importance of agonistic and affiliative behaviors in bovine social hierarchy [49–52], the calculated BI was used to establish a ranking from 1st to 14th, based on social hierarchy classification.

Parameters from the SNA, in association with weight, ADG, testosterone, BI, and ranking, were subjected to a principal component analysis (PCA; princomp{stats}'). First, on the biplot of the PCA ('fviz_pca{factoextra}'), confidence ellipses were prepared based on the distribution of the density of the bulls in each moment. Then, through a qualitative judgment, the distance between each ellipsis and their distribution in the four quadrants of the biplot were analyzed, and the distance between ellipses and their relationship with the origin (convergence between the x and y axes of ellipses).

A posteriori, to confirm and interpret inferences of the results, confirmatory statistical analyses were performed. Data distributions were analyzed through histograms ('hist{graphics}'), quantile–quantile plots ('qqnorm{stats}') and boxplots ('ggboxplot{ggpubr}'); graphically, all variables were defined as non-normal. Therefore, variables were compared over time (pre- vs post- castration) with paired two-tailed Wilcoxon tests ('wilcox.test{stats}').

The delta (Δ) of the ranking was extracted by subtracting the classification attributed post- minus pre-castration. Thus, individuals with ascension in the social hierarchy ranking post castration were considered Δ positive, whereas those that downgraded were Δ negative.

To further investigate which factors related to the castration influenced the downgrading of individuals in the ranking, two logistic multilinear regression was done for each moment ('glm{stats}'). For the pre-castration moment, the possibility of the ranking be considered as Δ positive or negative (dichotomic) was used as the dependent variable, whereas the weight, ADG, and testosterone concentration were used as independent variables. For the post-castration moment, the same parameter was used as the dependent variable, whereas the weight, ADG, surgery order, and inflammation level were used as independent variables.

If the independent variable (inflammation level) had a significant effect, a Spearman correlation was performed ('rcorr{Hmisc}') with the independent variable – possibility of ranking of Δ being positive or negative. Lastly, for ADG after the castration, bulls with social hierarchy rank with negative Δ were compared to positive Δ using a non-paired, two-tailed Wilcoxon test.

## Supplementary Information

**Additional file 1:Table S1.** Individual values for variables used in social net analyses for agonistic behavior (A), affiliative behavior (B) and hierarchy ranking, balance index (BI), testosterone, average daily weight gain (ADG), and scrotal circumference (C) before and after castration of Nelore Bulls. **Table S2.** Charge value, eigenvalue and variance of the principal component analyzes before and after castration of the Nelore bulls. In bold are charge values > 0.60 or < - 0.60.

## Data Availability

The datasets used and analyzed during the current study are available on supplementary material.
