# Supplementary material for: Wound inflammation post-orchiectomy affects the social dynamic of Nelore bulls
Source: BMC Vet Res. 2023 Jul 15;19:84. doi: 10.1186/s12917-023-03638-9 (PMC10349438; doi:10.1186/s12917-023-03638-9)
Supplement: Supplementary file 1 — Additional file 1:Table S1. Individual values for variables used in social net analyses for agonistic behavior (A), affiliative behavior (B) and hierarchy ranking, balance index (BI), testosterone, average daily weight gain (ADG), and scrotal circumference (C) before and after castration of Nelore Bulls. Table S2. Charge value, eigenvalue and variance of the principal component analyzes before and after castration of the Nelore bulls. In bold are charge values > 0.60 or < - 0.60. [file 12917_2023_3638_MOESM1_ESM.docx]

Table S1: Individual values for variables used in social net analyses for agonistic behavior (A), affiliative behavior (B) and hierarchy ranking, balance index (BI), testosterone, average daily weight gain (ADG), and scrotal circumference (C) before and after castration of Nelore Bulls.

| A |  |  |  |  |  |  |  |  |
| --- | --- | --- | --- | --- | --- | --- | --- | --- |
| Moment | Bull | Betweenness_AG_ | Closeness_AG_ | Degree ALL_AG_ | Degree IN_AG_ | Degree OUT_AG_ | Authors_AG_ | Receptors_AG_ |
| Pre-castration | 7 | 0 | 0.032258 | 1 | 1 | 0 | 2.15^-18^ | 1.85^-05^ |
|  | 5 | 20.13333 | 0.052632 | 10 | 6 | 4 | 0.000356 | 0.060368 |
|  | 10 | 1.740741 | 0.047619 | 7 | 5 | 2 | 0.003222 | 0.018203 |
|  | 9 | 4.256039 | 0.045455 | 42 | 21 | 21 | 0.102111 | 1 |
|  | 12 | 1.777778 | 0.041667 | 7 | 4 | 3 | 0.00354 | 0.208143 |
|  | 13 | 20.29553 | 0.052632 | 20 | 12 | 8 | 0.022297 | 0.145005 |
|  | 6 | 66.21164 | 0.0625 | 55 | 24 | 31 | 1 | 0.078663 |
|  | 1 | 11.58494 | 0.055556 | 14 | 6 | 8 | 0.10876 | 0.060398 |
|  | 4 | 14.77778 | 0.047619 | 9 | 5 | 4 | 0.019223 | 0.006606 |
|  | 11 | 11 | 0.041667 | 6 | 2 | 4 | 0.01338 | 0.052166 |
|  | 2 | 13.55556 | 0.052632 | 9 | 5 | 4 | 0.007884 | 0.002462 |
|  | 14 | 0.25 | 0.038462 | 6 | 1 | 5 | 0.023952 | 0.00116 |
|  | 3 | 1.416667 | 0.045455 | 7 | 6 | 1 | 0.00251 | 0.163582 |
|  | 8 | 0 | 0.035714 | 3 | 0 | 3 | 0.011353 | 1.00^-26^ |
|  |  |  |  |  |  |  |  |  |
| Post-castration | 3 | 0 | 0.038462 | 2 | 2 | 0 | 1.32^-17^ | 0.030425 |
|  | 8 | 0 | 0.03125 | 1 | 1 | 0 | 6.61^-18^ | 0.060767 |
|  | 9 | 0 | 0.037037 | 8 | 8 | 0 | 5.29^-17^ | 0.925394 |
|  | 13 | 0 | 0.035714 | 1 | 1 | 0 | 6.61^-18^ | 0.005365 |
|  | 7 | 28.7 | 0.04 | 7 | 4 | 3 | 0.127849 | 0.126975 |
|  | 14 | 39.975 | 0.05 | 16 | 7 | 9 | 0.338914 | 1 |
|  | 4 | 16.02778 | 0.045455 | 8 | 4 | 4 | 0.139767 | 0.648774 |
|  | 11 | 13.875 | 0.05 | 14 | 1 | 13 | 0.618413 | 0.02506 |
|  | 5 | 41.20833 | 0.0625 | 18 | 13 | 5 | 0.029921 | 0.965656 |
|  | 6 | 34 | 0.04 | 7 | 6 | 1 | 0.052387 | 0.407444 |
|  | 1 | 0 | 0.037037 | 3 | 2 | 1 | 0.069337 | 0.22176 |
|  | 2 | 42.69231 | 0.047619 | 12 | 7 | 5 | 0.340479 | 0.729587 |
|  | 12 | 30.52158 | 0.055556 | 21 | 3 | 18 | 1 | 0.282527 |
|  | 10 | 0 | 0.04 | 4 | 2 | 2 | 0.138674 | 0.358596 |

| B |  |  |  |  |  |  |  |  |
| --- | --- | --- | --- | --- | --- | --- | --- | --- |
| Moment | Bull | Betweenness_AF_ | Closeness_AF_ | Degree ALL_AF_ | Degree IN_AF_ | Degree OUT_AF_ | Authors_AF_ | Receptors_AF_ |
| Pre-castration | 7 | 8.416667 | 0.026316 | 7 | 5 | 2 | 0.000924 | 0.228611 |
|  | 5 | 0 | 0.022727 | 2 | 0 | 2 | 0.000924 | 0 |
|  | 10 | 8.5 | 0.025 | 5 | 3 | 2 | 0.01772 | 0.002504 |
|  | 9 | 13.74737 | 0.025 | 31 | 12 | 19 | 1 | 0.002526 |
|  | 12 | 0 | 0.008264 | 2 | 1 | 1 | 1.52^-17^ | 0 |
|  | 13 | 1.333333 | 0.023256 | 7 | 4 | 3 | 0.00112 | 0.444444 |
|  | 6 | 2 | 0.02381 | 19 | 9 | 10 | 0.002255 | 1 |
|  | 1 | 0 | 0 | 0 | 0 | 0 | 0 | 0 |
|  | 4 | 5.7 | 0.025 | 11 | 7 | 4 | 0.019093 | 0.009525 |
|  | 11 | 0 | 0 | 0 | 0 | 0 | 0 | 0 |
|  | 2 | 0 | 0.020833 | 5 | 4 | 1 | 0.000194 | 0.444444 |
|  | 14 | 0 | 0.008264 | 2 | 1 | 1 | 1.52^-17^ | 0 |
|  | 3 | 0 | 0.020833 | 1 | 1 | 0 | 4.83^-18^ | 0.001969 |
|  | 8 | 5.302632 | 0.025641 | 10 | 4 | 6 | 0.020688 | 0.006615 |
|  |  |  |  |  |  |  |  |  |
| Post-castration | 3 | 0 | 0 | 0 | 0 | 0 | 0 | 0 |
|  | 8 | 0 | 0 | 0 | 0 | 0 | 0 | 0.00 |
|  | 9 | 0 | 0.05 | 3 | 3 | 0 | 0 | 0.120224 |
|  | 13 | 0 | 0.038462 | 1 | 1 | 0 | 0 | 0.1482 |
|  | 7 | 0 | 0.052632 | 2 | 0 | 2 | 0.25 | 1.68^-17^ |
|  | 14 | 0 | 0.045455 | 2 | 0 | 2 | 0.030056 | 1.68^-17^ |
|  | 4 | 0 | 0.041667 | 1 | 0 | 1 | 1.83^-17^ | 8.39^-18^ |
|  | 11 | 0 | 0.047619 | 1 | 0 | 1 | 0.241068 | 8.39^-18^ |
|  | 5 | 1 | 0.055556 | 6 | 1 | 5 | 1 | 0.03705 |
|  | 6 | 0 | 0 | 0 | 0 | 0 | 0 | 0.00 |
|  | 1 | 0 | 0 | 0 | 0 | 0 | 0 | 0 |
|  | 2 | 5 | 0.0625 | 3 | 2 | 1 | 0.028982 | 4.18^-17^ |
|  | 12 | 2 | 0.058824 | 5 | 1 | 4 | 0.752187 | 0.004454 |
|  | 10 | 8 | 0.076923 | 10 | 9 | 1 | 3.93^-17^ | 1 |

| C |  |  |  |  |  |
| --- | --- | --- | --- | --- | --- |
| Moment | Bulls | Testosterone | Weight | ADG | Scrotal circumference |
| Pre-castration | 7 | 15,600.6 | 416 | -4.12 | 31 |
|  | 5 | 2,449.4 | 418 | -2.69 | 31 |
|  | 10 | 4,758.6 | 417 | -1.48 | 31 |
|  | 9 | 8,661.1 | 447 | -0.92 | 32 |
|  | 12 | 6,43.9 | 464 | -1.75 | 32 |
|  | 13 | 7,741.3 | 386 | -1.89 | 31 |
|  | 6 | 14,532.9 | 449 | -1.43 | 33 |
|  | 1 | 8,219.3 | 460 | -2.81 | 35 |
|  | 4 | 1,212.1 | 422 | -1.56 | 33 |
|  | 11 | 1,407.9 | 452 | -1.72 | 33 |
|  | 2 | 1,307.8 | 418 | -1.77 | 33 |
|  | 14 | 899.4 | 422 | -6.56 | 35 |
|  | 3 | 4,610.2 | 483 | -1.52 | 32 |
|  | 8 | 1,015.8 | 442 | -2.05 | 32 |
|  |  |  |  |  |  |
| Post-castration | 3 | 3.9 | 592 | 0.99 | NA |
|  | 8 | 3.9 | 518 | 0.73 | NA |
|  | 9 | 3.9 | 508 | 0.63 | NA |
|  | 13 | 3.9 | 464 | 0.53 | NA |
|  | 7 | 3.9 | 485 | 0.47 | NA |
|  | 14 | 3.9 | 534 | 0.26 | NA |
|  | 4 | 3.9 | 483 | -0.53 | NA |
|  | 11 | 3.9 | 534 | 0.26 | NA |
|  | 5 | 3.9 | 500 | 1.39 | NA |
|  | 6 | 3.9 | 558 | 0.47 | NA |
|  | 1 | 3.9 | 576 | 0.46 | NA |
|  | 2 | 3.9 | 524 | 0.4 | NA |
|  | 12 | 3.9 | 584 | 0 | NA |
|  | 10 | 3.9 | 472 | 0.47 | NA |

NA = non-applicable value.

Table S2: Charge value, eigenvalue and variance of the principal component analyzes before and after castration of the Nelore bulls. In bold are charge values > 0.60 or < - 0.60

| Variable | Dimmension 1 | Dimmension 2 |
| --- | --- | --- |
| Betweenness_AG_ | 0.54 | 0.57 |
| Closeness_AG_ | 0.61 | 0.39 |
| Degree ALL_AG_ | 0.94 | 0.14 |
| Degree IN_AG_ | 0.88 | 0.06 |
| Degree OUT_AG_ | 0.87 | 0.19 |
| Authors_AG_ | 0.55 | 0.52 |
| Receptors_AG_ | 0.35 | 0.46 |
| Betweenness_AF_ | 0.52 | -0.49 |
| Closeness_AF_ | 0.29 | 0.41 |
| Degree ALL_AF_ | 0.87 | -0.34 |
| Degree IN_AF_ | 0.75 | -0.47 |
| Degree OUT_AF_ | 0.87 | -0.19 |
| Authors_AF_ | 0.54 | 0.32 |
| Receptors_AF_ | 0.47 | -0.17 |
| Testosterone | 0.57 | -0.52 |
| Weight | -0.23 | 0.76 |
| ADG | -0.05 | 0.67 |
| BI | -0.06 | -0.28 |
| Ranking | -0.03 | -0.21 |
|  |  |  |
| Eigenvalue | 6.85 | 3.36 |
| Variance | 36.03 | 17.67 |
